# Spontaneous Pneumobilia: Not So Benign

**DOI:** 10.7759/cureus.14486

**Published:** 2021-04-14

**Authors:** Abdul Ahad E Sheikh, khalid H Ahmed, Sreekant Avula, Niraj J Shah, Mark M Aloysius

**Affiliations:** 1 Internal Medicine, The Wright Center for Graduate Medical Education, Scranton, USA; 2 Gastroenterology, University of Mississippi Medical Center, Jackson, USA

**Keywords:** pneumobilia, cholangitis, sponatenous pneumobilia, increased intraabdominal pressure

## Abstract

Pneumobilia is defined as air within the biliary system. It is usually caused by an abnormal connection between the biliary gastrointestinal tracts. Persistent asymptomatic pneumobilia is a rare occurrence and is generally considered a benign finding on imaging. Herein, we present a case of an 87-year-old male with long-standing pneumobilia of no identifiable cause who eventually developed Klebsiella cholangitis and bacteremia. In our report, we attempt to elucidate the causes of spontaneous pneumobilia and discuss its significance in the context of increased intraabdominal pressure.

## Introduction

Most cases of pneumobilia are iatrogenic and easily identified on imaging. Some of the non-iatrogenic causes include emphysematous cholecystitis, ascending cholangitis (usually with gas-forming organisms), spontaneous biliary-enteric fistula, biliary bronchopleural fistula, incompetent sphincter of Oddi, and visceral blunt trauma [1]. Timely awareness of pneumobilia etiology is imperative to enable prompt accurate treatment to avert catastrophic sequelae. We present a unique case of a patient developing acute cholangitis in the setting of long-standing spontaneous pneumobilia and increased intraabdominal pressure. This has not been previously reported in the literature.

## Case presentation

An 87-year-old male with a past medical history notable for small bowel resection and anastomosis following obstruction, paroxysmal atrial fibrillation, basal cell carcinoma of the skin, and open cholecystectomy about 30 years ago, presented with complaints of generalized weakness and abdominal discomfort for two weeks prior to presentation. The abdominal discomfort was mostly after meals and progressively worsened. This was accompanied by high-grade fevers, nausea, and generalized weakness prompting the ED visit. Previously, this patient had three admissions over the last couple of years with abdominal pain, nausea, and vomiting, diagnosed as small bowel obstruction secondary to adhesions, managed conservatively to spontaneous full resolution, except for the last admission five months ago during which he required a repeat laparotomy and lysis of adhesions. During each of his previous admissions, pneumobilia was noted on CT scans of the abdomen, despite the lack of any symptoms related to cholangitis or sepsis. 

**Figure 1:**
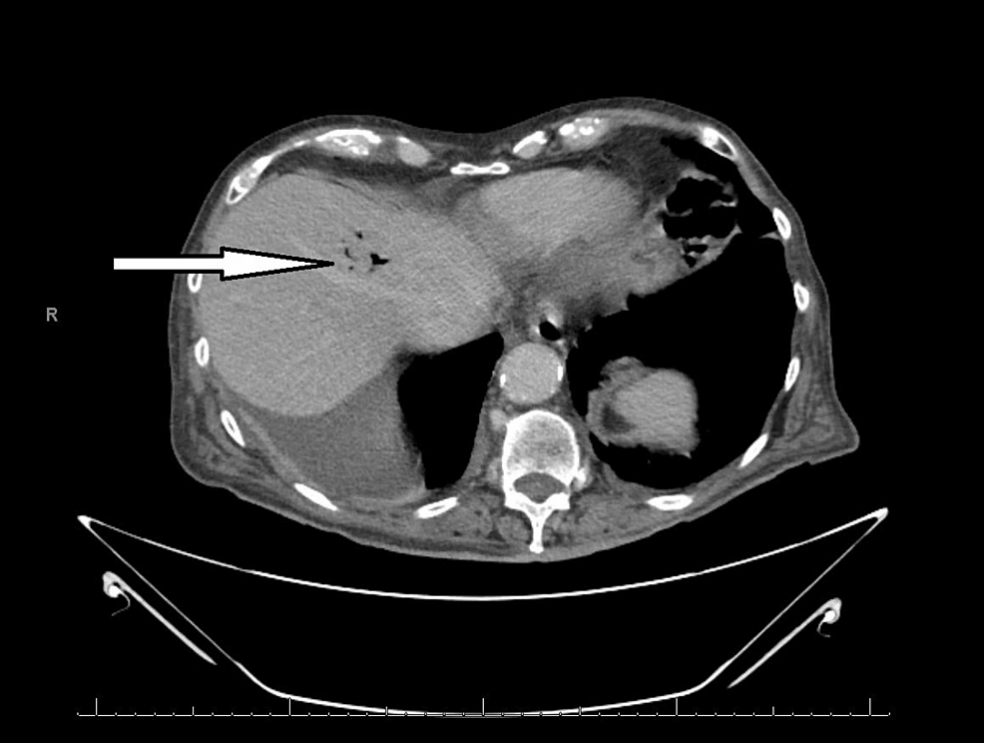
Axial plane CT abdomen and pelvis obtained shortly after admission showing branching air in the left hepatic lobe (arrow) which could represent pneumobilia.

**Figure 2:**
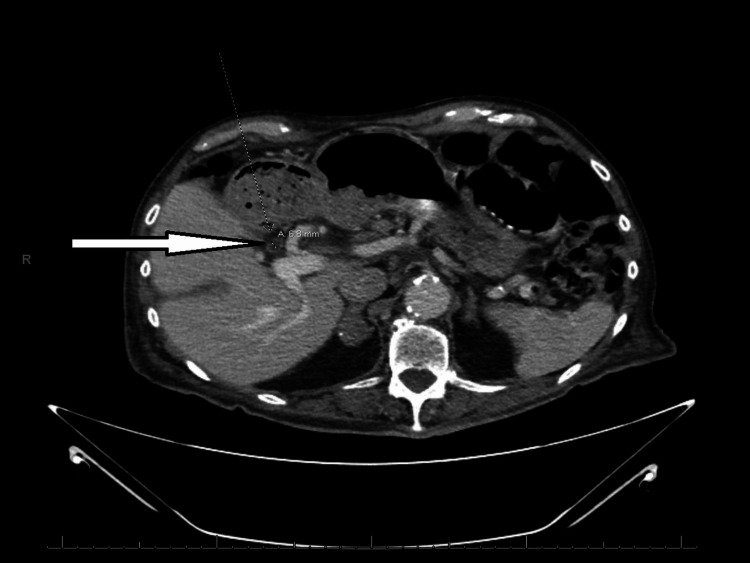
CT abdomen and pelvis on presentation showing thickening duct wall (arrow), which is non-specific but could represent cholangitis in the correct clinical setting.

**Figure 3:**
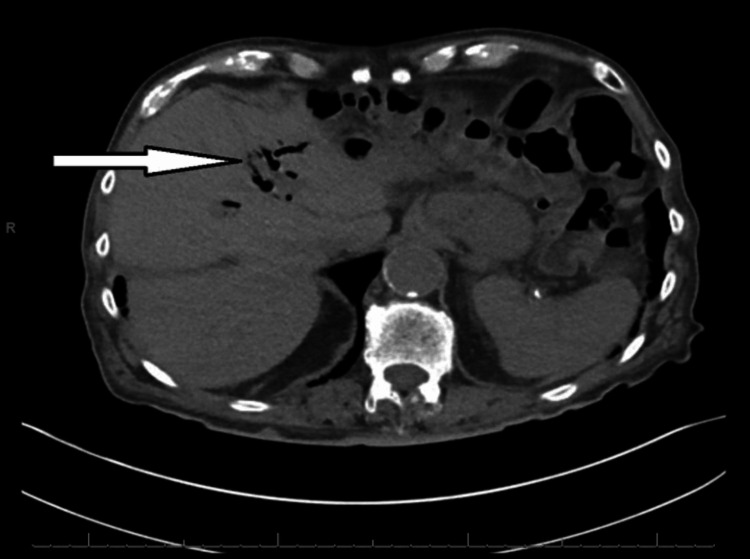
Axial plane of CT abdomen and pelvis two years prior to presentation with cholangitis and subsequent ERCP revealing pneumobilia (arrow).

On physical examination, this elderly male was found to be in moderate distress, with a temperature of 100.2°F, respiratory rate of 17/min, blood pressure (BP) of 106/64 mmHg, heart rate of 79/min, and SpO2 of 97% on room air. On auscultation, he had dual heart sounds with a holosystolic murmur noted at the apex. Breath sounds were diminished bilaterally but equal with no crackles or rhonchi heard. The abdomen was soft, non-distended but slightly tender. On laboratory testing, the patient had a white blood cell count (WBC) of 7.13 K/uL, hemoglobin of 14.6 g/dL, and a platelet count of 147,000 which were all within normal limits. The basic metabolic panel revealed creatinine of 1.0 mg/dL, electrolytes were within normal limits except potassium of 3.3 mmol/L. Liver chemistries revealed an elevated alanine aminotransferase (ALT) of 425 U/L, aspartate aminotransferase (AST) of 898 U/L, total bilirubin 2.5 mg/dL, and alkaline phosphatase (ALP) 612 U/L. Lactic acid was found to be at 2.5. CT scan of the abdomen without contrast showed branching air throughout the central aspect of the left hepatic lobe along with mild wall thickening of the common hepatic duct as shown in Figures 1, 2. This finding of pneumobilia was consistently unchanged from previous CT scans over the last two years as shown in Figure 3 below.

**Figure 4:**
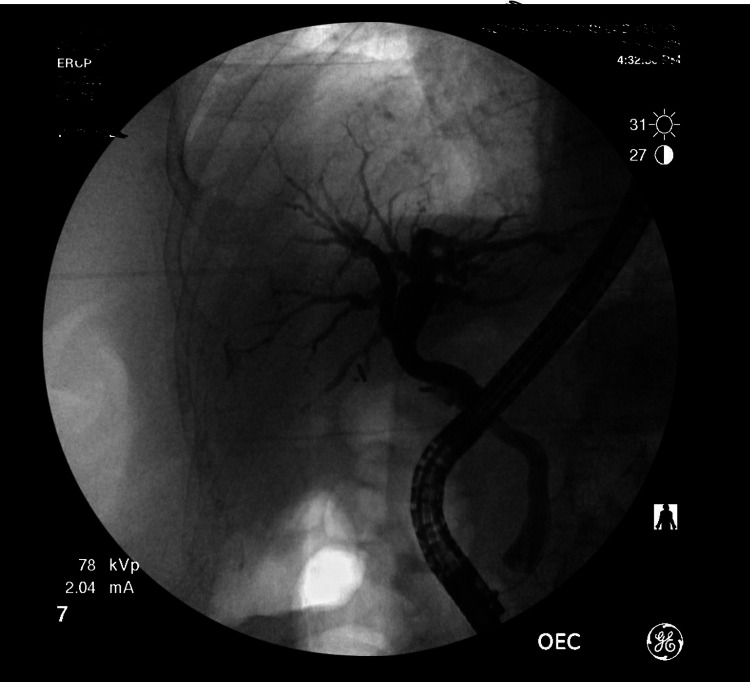
ERCP showing no filling defects following sphincterotomy. Endoscopic retrograde cholangiopancreatography (ERCP)

A provisional diagnosis of sepsis secondary to cholangitis was made and the patient was started on IV fluids and antibiotics. He was found to be bacteremic with blood cultures growing Klebsiella. Gastroenterology was consulted and an endoscopic retrograde cholangiopancreatography (ERCP) was performed, which showed dilation of the common bile duct as shown in Figure 4. A 4 mm sphincterotomy was performed and numerous small biliary stones and sludge were removed along with occasional pus. A plastic stent was also placed in the common bile duct. 

Initially, the patient's pneumobilia was attributed to his current cholangitis, however, the patient had pneumobilia since at least 2018. No choledochoduodenal fistula, choledochoduodenostomy, or evidence of the previous sphincterotomy was observed during the ERCP. Antibiotics were deescalated to an oral regimen, and the patient was discharged home following stabilization.

## Discussion

Intra-biliary air is an ominous discovery and life-threatening causes need to be excluded expeditiously [1]. Pneumobilia in a patient who is clinically unstable is an emergency. The etiologies are related to either some form of a biliary-enteric fistula or emphysematous cholecystitis [2,3]. The additional finding of air within the portal venous system indicates bowel wall ischemia, gas gangrene, or pyogenic liver abscess [4,5]. There have been sporadic reports of pneumobilia caused by bacterial cholangitis and parasites such as Echinococcus [1,6]. However, despite its description in case reports, the true prevalence of spontaneous pneumobilia from sphincter of Oddi incompetence is unknown [1].

We describe a rare case of spontaneous persistent pneumobilia preceding acute cholangitis in a patient with multiple episodes of bowel obstruction over a few years and review other etiologies of this radiological finding which should trigger a comprehensive evaluation and management strategy. Spontaneous pneumobilia is an uncommon incidental finding and may be a harbinger of cholangitis. This is plausible especially if the pneumobilia and bile reflex are caused by an incompetent sphincter of Oddi exacerbated by increased enteric intraluminal pressure from a recurrent small bowel obstruction. There have been numerous reports of pneumobilia caused by blunt abdominal trauma with an intact sphincter of Oddi [7-13].

More benign causes of pneumobilia are post-cholecystectomy, post ERCP, or following percutaneous transhepatic cholangiography (PTC) [14,15]. However, these should resolve spontaneously in weeks to months unless the sphincter of Oddi is incompetent, in which case pneumobilia can persist for years as seen in our patient. Of note, he underwent cholecystectomy 30 years ago which did not reveal a biliary-enteric fistula. There was no evidence of any biliary tract injury during his previous two laparotomies with adhesiolysis for small bowel obstruction. There was no evidence of clinical or radiological findings of bowel ischemia or a hepatic abscess during any of his hospital admissions for recurrent small bowel obstruction. However, his pneumobilia was a persistent finding during his multiple hospital admissions for small bowel obstruction, prior to the development of cholangitis. The patient underwent ERCP during which purulent material was drained from the common bile duct, and there was no extraneous communication with bowel indicative of any biliary-enteric fistula seen in cholangiography. A sphincterotomy was performed during the procedure. 

To our knowledge, this report appears to be the first to document a case of persistent pneumobilia preceding acute cholangitis. The absence of gallbladder makes retrograde enteric reflux into the common bile duct associated with recurrent intraluminal pressure from obstruction a strong etiological plausibility in our patient, although sphincter pressure was not measured during ERCP. This case highlights pneumobilia as a potential premorbid incidental finding, that may be a harbinger of cholangitis in the setting of increased enteric intraluminal pressure.

## Conclusions

Spontaneous pneumobilia is a benign entity. However, its risk of cholangitis in combination with recurrent increased intraabdominal pressure has not been documented before. Our case illustrates that such an occurrence is a possibility and clinical vigilance is required when dealing with this combination. Such patients need to be followed up regularly with a low threshold for diagnosis of cholangitis if symptoms arise so early intervention can reduce morbidity and mortality. Persistent spontaneous pneumobilia in the setting of recurrent bowel obstructions may be a harbinger of acute cholangitis.
